# Feasibility of a Community Healthy Eating and Cooking Intervention Featuring Traditional African Caribbean Foods from Participant and Staff Perspectives

**DOI:** 10.3390/nu15173758

**Published:** 2023-08-28

**Authors:** Sally G. Moore, Aashna Kundra, Peter Ho, Esther Bissell, Tanefa Apekey

**Affiliations:** 1 School of Food Science and Nutrition, University of Leeds, Leeds LS2 9JT, UK; a.k.kundra@leeds.ac.uk (A.K.); p.ho@leeds.ac.uk (P.H.); e.c.bissell@leeds.ac.uk (E.B.); 2 School of Health and Related Research, Sheffield S1 4DA, UK; t.apekey@sheffield.ac.uk

**Keywords:** culturally adapted, intervention, healthy eating, health promotion, food-based dietary guidance, recipes, co-development, cooking skills, African Caribbean

## Abstract

Culturally appropriate healthy eating resources are intended to help people from different ethnic backgrounds consume diets reflecting government dietary recommendations, yet evidence on use in the target groups is lacking. This study evaluated the feasibility of a new brief culturally appropriate community intervention that aimed to introduce food-based healthy eating and recipe resources featuring African Caribbean foods, which were recently co-developed with people from these ethnic backgrounds. Working with a community organization in the UK, a single-arm study was used to collect verbal data from participants and staff on the acceptability of intervention whilst knowledge, skills and behaviours related to healthy eating were evaluated using pre-, post- and follow-up questionnaires. A total of 30 participants were recruited, and 22 completed all three questionnaires; who were mostly female aged 55 years+ (n = 17) and of African Caribbean ethnicity (45%, n = 10), with 32% (n = 7) reporting no educational attainment. At post-intervention and follow-up, most participants reported high satisfaction (n = 21, 95%) with the intervention sessions and high levels of confidence in using the resources at home within budget. The number of participants who were familiar with the healthy eating guidance featuring Caribbean foods increased from pre- (36%, n = 8) to post-intervention/follow-up (n = 22, 100%) (*p* < 0.05). Findings suggest the intervention is feasible in a community setting and could help increase awareness and use of culturally appropriate healthy eating guidance amongst a diverse group.

## 1. Introduction

In the UK, there is a need to tackle health inequalities in health and diet across different population groups since higher levels of cardiovascular disease, type 2 diabetes and obesity are experienced by people of different ethnicities, including those of African Caribbean backgrounds, compared to Caucasian populations [1]. Poor health outcomes are also related to dietary intake (i.e., of energy, saturates, sugars, salt, and fibre), which are known to fall outside of Government dietary recommendations for most people in the UK [2], including people of African Caribbean ethnicities [3,4,5]. In the UK, health outcomes, obesity levels, and dietary quality are all expected to worsen in the current economic crisis [6].

One way to support people with healthier eating is to provide guidance on how they could incorporate this into their daily lives by using practical “food-based” resources featuring commonly consumed familiar foods. This approach is also suggested ina recently published guidance collection for UK health professionals aimed at improving and personalizing care and population health [7]. As such, a common feature of healthier eating initiatives, such as those available from the UK NHS Healthier Families campaign, is to provide food-based suggestions for diets and recipes that deliver lower levels of energy (calories) or nutrients of public health concern [8]. Healthier recipe preparation and cooking at home can also provide budget-friendly, less-processed foods and respond to the current cost of living crisis and the health concerns around the consumption of “ultra-processed” foods [9,10]. However, there is presently a lack of culturally appropriate resources and interventions targeting food choices and healthy eating for people from different ethnicities. Diversification of nutrition advice and improving the cultural appropriateness of practical food-based resources is a currently recognised need for nutrition and health professionals [11].

Culturally appropriate, healthier eating interventions and food-based resources are ideally specifically adapted or tailored to meet the needs of different populations [12]. As such, these may help address some of the barriers around food choice and preparation reported by people of different ethnicities. For example, in the UK, there are now new culturally adapted versions of the Eat Well Guide, which illustrate UK Government dietary recommendations using images of specific traditional foods commonly consumed by people from different ethnicities, including African Caribbean backgrounds [13]. In addition, co-development involving people of African Caribbean ethnicities has been used to create versions of healthier recipes of traditional foods and dishes that deliver improved levels of key nutrients of public health concern (i.e., lower levels of salt, saturated fat, and sugars) [14].

Evaluation of such interventions to determine how people and health professionals receive and can use such resources to enable impact in real-life diets and health outcomes is lacking. Such evidence can also further inform culturally competent, evidenced-based professional practice and policy that aims to address or reduce inequalities in public health. Therefore, the aim of the current study was to determine the feasibility of undertaking a community multicultural healthy eating education and cooking intervention featuring traditional African Caribbean foods at a community organization by evaluating service users’ and staff perceptions of the acceptability and relevance of using resources in real life/practice. The second aim was to evaluate the potential impact of the intervention and resources on participant’s food and cooking confidence, knowledge, and behaviours.

## 2. Materials and Methods

### 2.1. Study Design and Ethics

This feasibility evaluation was designed to establish if the intervention is practical to conduct, acceptable to participants and staff, and to identify any aspects requiring modification before further future testing of its effectiveness [15]. A single group pre- (immediately before the first intervention session), post- (immediately after the second session, 2–3 weeks later), and follow-up (one month later) design was used, with both quantitative and qualitative elements. The study design was based on best practice in designing feasibility studies [15], in line with the existing community partnership, inclusive of community service users from various ethnic backgrounds and the available research resources. The study was conducted in accordance with the Declaration of Helsinki and the University of Leeds AREA ethical approval (AREA FREC 2023-0377-331). All service users and staff were provided with a study information sheet during the recruitment stage and were required to provide informed written consent before participation.

### 2.2. Intervention Development and Delivery

**Figure 1:**
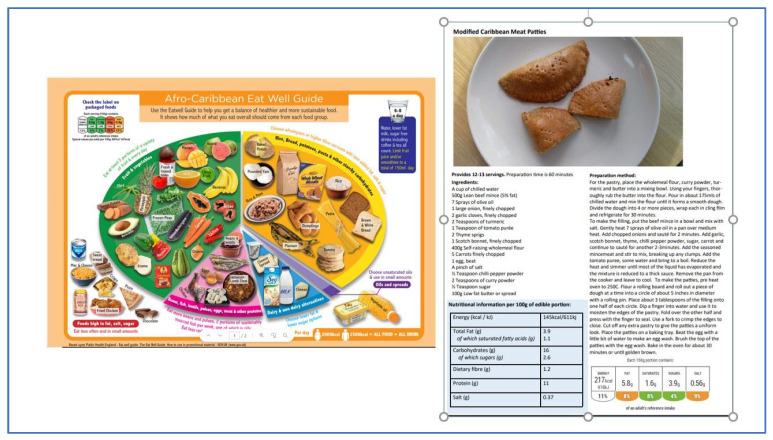
The two codeveloped food-based resources on healthy eating used in the intervention session. (**Left**) The African Caribbean Eat Well Guide used in session 1. (**Right**) One of the 12 healthier traditional recipe resources (Meat Patties) used in session 2.

The new two-part brief intervention aimed to support people who identified as being from African Caribbean ethnic backgrounds and those who commonly consumed traditional foods to choose, prepare and consume a healthy balanced diet featuring these popular culturally appropriate foods. Intervention development was based on two recently co-developed food-based healthy eating resources: the African Caribbean Eat Well Guide [13] and the healthier African Caribbean recipe resources [14] (Figure 1). Intervention development was based on promoting knowledge, skills and behaviours amongst participants [16], reflecting an enabling empowerment approach to culturally adapted interventions [17]. Intervention development and session format also incorporated input from staff at the community organization, which served service users from diverse groups, including people of first- and second-generation African Caribbean ethnicities. Across the two sessions, the intervention objectives were to (1) introduce participants to the two new food-based healthy eating resources, (2) contextualize these in healthy eating recommendations, and (3) provide hands-on experience preparing, cooking, and tasting the recipes.

The first intervention session (1 h) was conducted in person as a discussion group and introduced the two new food-based resources on healthy eating featuring traditional African Caribbean foods and contextualised them within UK healthy eating food-based guidance (i.e., “five-a-day fruit and vegetables”, “use of traffic lights to choose foods lower in fat sugar salt” etc.) [18]. Hard copies of the new African Caribbean Eat Well Guide [13] were given to all participants to encourage a discussion with real-life food products and reference to the “daily allowances” for salt, sugar, and saturated fat before introducing (with videos) and providing hard copies of the healthier recipes featuring traditional African Caribbean foods. These recipe resources are freely available online (see Foodwiseleeds.org (accessed on 1 December 2022) and include traditional dishes such as Meat Patties, Jerk Chicken and Cod Fish Fritters [14]. The second (3 h) cooking session provided all participants with opportunities to experience cooking, preparing and eating the healthier traditional recipes, with a focus on both cooking skills and healthier alternative ingredients. The intervention was named “Healthy Eating with African Caribbean Foods”, which appeared in recruitment adverts and communications. Each of the two intervention sessions was duplicated to accommodate up to 40 people in the available room space from February to March 2023. Sessions were delivered by a registered dietitian (SM) and nutritionist (AK) and community staff members who were experienced in leading group-based cooking activities.

### 2.3. Recruitment of Community Service Users and Staff

Participant recruitment was facilitated by community centre staff who contacted all their (n = 100) service users via email or text messages using permitted database information. In addition, recruitment posters were physically displayed around the community. Due to the need to be inclusive of all existing community service users and all ethnic backgrounds, no one was excluded based on ethnicity or other characteristics. All community centre staff whose roles were known to involve supporting the health of service users (n = 17) were invited by email to participate in an in-person or remote (online) staff session. The staff session was hosted by the researchers to showcase the intervention and resources and to invite staff to openly discuss their perceptions of these.

Following participant consent, pre-session questionnaires were provided as hard copies for participants to complete at the start of the first session, and post-session questionnaires were given out immediately after the second (cooking) session (2–3 weeks later). Researchers assisted one participant in completing the questionnaires as requested. To enable follow-up data collection, a coffee morning was held one month later, during which participants received a £25 retail voucher in appreciation for their participation in the study. Staff participants were provided with an online questionnaire following their session, and given a £15 retail voucher in appreciation for their participation.

### 2.4. Evaluation of Participant Perceptions of the Intervention, Including Acceptability and Relevance to Real Life

During their development, all three pre-, post-, and follow-up questionnaires were reviewed by staff at the community centre, whose feedback on these reflected prior knowledge and literacy needs of service users (Supplementary Information). Feedback was used to shorten the length of the questionnaire and revise some minor question wording and font size/presentation of questions for readability. Pre-intervention questionnaires (20 items) included information collection on participant demographics, educational attainment, weekly food spending and where food was obtained from, as well as if advice on diet had been received from a health professional. Information on allergies was also collected and used to inform the selection of 4–5 recipes for cooking in the second intervention session. Pre-intervention questionnaires also asked participants what they hoped to “get out” of the sessions (open text box). Participants’ verbal feedback and perceptions of the new intervention were invited and captured via audio recording during the sessions (MS Teams).

Post-intervention and follow-up questionnaires also asked participants for their perceptions of the acceptability of the intervention sessions, using a five-point category scale to rate their satisfaction with several aspects of the intervention sessions (1 = dissatisfied, 5 = very satisfied) and space for comments on the intervention and suggestions for improvement. A total of three question items were adapted by the researchers to assess participants’ familiarity with the new African Caribbean Eat Well Guide and if they were aware of the healthy eating guidance incorporating African Caribbean foods, the healthier recipe resources and daily allowances (with yes/no responses) [19]. The pre- and post-questionnaires also objectively assessed participants’ knowledge of specific aspects of healthy eating and recommended daily allowances (i.e., of sugars, salt, fruit, and vegetables) using multiple choice answer options as used previously [20,21].

Pre-, post- and follow-up questionnaires used a five-point category scale to assess the importance of recipe use (i.e., 1 = not at all important, 5 = extremely important) adapted from a questionnaire validated to assess cooking skills amongst low-income populations [22]. Participants were also asked to rate their possession of recipe-related skills, including “ability to follow recipes”, “plan meals and shopping” etc., using a five-point Likert scale (i.e., 1 = completely disagree, 5 = completely agree) [22]. At post-intervention and follow-up, three items were adapted to evaluate participants’ levels of confidence when specifically using the healthier African Caribbean recipes “in day-to-day cooking”, “using healthier ingredients,” and “cooking healthier recipes within my food budget”, using with a 5 point scale (1 = not at all confident, 5 = extremely confident). Finally, participants were asked in post- and follow-up questionnaires about their anticipated or actual use of the resources selecting from options (i.e., shared recipes with family and friends, shared African Caribbean health eating guidance with friends and family, N/A, Other), and if there was anything they would or had changed (open text box).

### 2.5. Participants Reported Compliance with Eat Well Guide Dietary Recommendations

Pre-, post-and follow-up questionnaires also evaluated participants’ self-reported dietary compliance with nine aspects of the Eat Well Guide dietary recommendations (i.e., consuming five portions of fruit and vegetables daily, consuming lower-fat dairy products or alternatives, including fish, etc.) [18]. The nine items used a five-point Likert scale (1 = completely disagree, 5 = completely agree) based on the Eat Well Guide (EWG) questions in the Consumer and Healthy Eating and Wellbeing collection previously validated for use with consumers [23]. In addition, a seven-item multiple-select question was also included to assess the participants’ perceived personal healthy eating practices (HEP) (i.e., drinking water every day, maintaining a balanced diet, avoiding foods high in sugar).

### 2.6. Staff Perceptions of the Intervention and Evaluation of the Potential to Impact on Practice

At the staff learning session, staff were invited to offer their thoughts on the intervention activities, content, sessions and resources, and verbal comments were captured via audio recording (MS Teams) and via a hard copy questionnaire. The staff questionnaire was developed by researchers to capture staff members’ current roles and backgrounds as well as any prior training in nutrition. A five-point category scale (1 = confident, 5 = not confident) was used to evaluate levels of staff confidence when providing culturally appropriate nutrition-related guidance to people from diverse backgrounds, with wording adapted from a basic self-assessment checklist of cultural competence [24].

### 2.7. Data Analysis

#### 2.7.1. Evaluation of the Feasibility of the Intervention

The feasibility of the intervention was evaluated using participant attendance, open-text questionnaire responses, and levels of participant satisfaction with various aspects of the intervention and sessions. For summary analysis, satisfaction ratings were collapsed as “not satisfied” (dissatisfied and neither satisfied nor unsatisfied) and “satisfied” (satisfied/very satisfied). Qualitative insight was also used to evaluate the perceived acceptability and relevance of the intervention from staff and participant perspectives. Automatically generated transcripts (n = 700 pages each) of each of the staff and participant session recordings (MS Teams), as well as written comments on questionnaires, were first read entirely by the researcher (AK) to familiarise with the text and who then provisionally summarized and grouped comments related to the any of following three preconceived themes, based on the aims of the intervention and feasibility study; (a) the acceptability and positive/negative perceptions on the intervention and resources (b) the application of learnings in real life/practice (c) areas of uncertainty and suggestions for future improvementThis deductive approach was based on best practices in qualitative research aiming to enhance rigour [25]. Furthermore, the reliability of data interpretation was also enhanced by using an independent review of the initial allocation undertaken by a second experienced researcher, also involved in the sessions (SM) [25,26]. Two queries were resolved by consensus. Illustrative quotes are presented to evidence feasibility, and a summary of participants’ comments is provided using a generated word cloud [27].

#### 2.7.2. Evaluation of the Potential for the Intervention to Impact Participants’ Healthy Eating-Related Skills, Knowledge and Behaviours

Each participant’s (hard copy) questionnaire responses were manually inputted into online surveys (JISC) by the researcher (AK) before exporting these into an Excel spreadsheet for onward analysis in R Studio (version 4.3.0) [27]. Participants who had provided responses for all three (pre-, post- and follow-up) questionnaires were included in the data analysis, with any missing questionnaire or item responses also indicated. Descriptive statistics of responses to questionnaire items evaluating recipe use and confidence, which used category scales, were calculated using the median and Inter Quartile Range (IQR) (i.e., 25th and 75th percentile values). Differences in participants’ responses to such items at pre-, post-, and follow-up were evaluated using a non-parametric Kruskal–Wallis test for scaled responses. In addition, the participants’ correct responses to the three knowledge MCQ quiz questions at pre- and post-intervention were calculated as a percentage proportion. Differences in proportions of correct/incorrect answers at pre- vs. post-time points were analyzed using Fischer’s exact test of independence.

Analysis of participants’ self-reported compliance with Eat Well Guide (EWG) recommendations included summarizing both the EWG and HEP measures from the perspective of modern test theory [28,29]. A Rasch measure (i.e., total score), composed of the nine EWG items, reflected the degree of perceived compliance to the Eatwell guide and was estimated by fitting a Rasch Rating Scale model using the Joint Maximum Likelihood Estimation (JMLE) method [29]. The EWG Rasch measure was then compared against a measure of adherence to healthy eating practices (HEP) by fitting the dichotomous Rasch model to responses from the HEP (multiple-choice questions on healthy eating practices). Both Rasch models were fitted using Winsteps [28] and Rasch estimates were rescored to 0–100 from logits after following standard procedures for checking model and item fit, proper functioning of the rating scale and unidimensionality of a Rasch model [29]. Statistical different levels of performance for person and item estimates were determined from a score-to-measure conversion table and represented as 95% confidence interval lines on a plot comparing the EWG Rasch estimates with the HEP Rasch estimates.

Post-hoc analysis was used to explore any differences in participants’ knowledge, perceived EWG compliance, satisfaction and awareness of recipes, etc., between the following sub-groups: Educational attainment (i.e., none, secondary/GSCE/O levels, and tertiary/higher education) and ethnic backgrounds (i.e., those from African/Caribbean backgrounds or other). For all tests, statistical significance was accepted at *p* < 0.05.

## 3. Results

### 3.1. Sample Characteristics

A total of 30 participants were recruited to participate in this intervention, of which 28 provided consent and attended the first intervention sessions and completed the pre-intervention questionnaire (Table 1), 24 attended the second (cooking) sessions, and 25 attended the follow-up coffee morning. Reasons for non-attendance were verbally reported by participants and included other personal commitments. Completed pre-, post-, and follow-up questionnaires were received from a total of 22 participants, who were mostly female (n = 18, 82%), aged 56–65 years (n = 9, 41%), and of African Caribbean ethnicities (n = 10, 45%), with 33% (n = 7) having no formal education. (Table 1). No significant differences in sample characteristics were found between consenting and completing participants (Table 1), and further analysis was undertaken using data from the 22 participants who had submitted all three questionnaires.

### 3.2. Participant Perceptions of the Intervention

**Figure 2:**
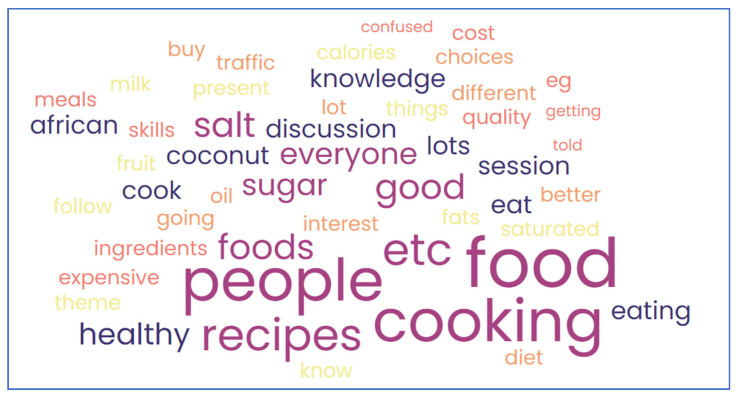
Word cloud summary of participant verbal feedback on the intervention.

Qualitative insight from pre-intervention questionnaires included what participants hoped to “get out” of the sessions. This included to “*think more about what I eat*” and “*to learn new cooking methods that I can include within my food budget and still enjoy traditional food from the Caribbean*”; or gaining “*information on how African Caribbean foods can improve my diet/health*” and learn “*other ways of cooking meals*”. Participant views verbalized during the intervention sessions included surprise at the daily allowance quantities (i.e., for salt) “*…6 g seems not a lot*” (Figure 2). Barriers to the use of recipes in real life were also raised, including cost; “*Most of the recipes require using 1–2 tablespoons of coconut milk, but since I live alone, if I open the tin and use only a little, the rest goes to waste hence I do not usually make recipes like those*”.

**Figure 3:**
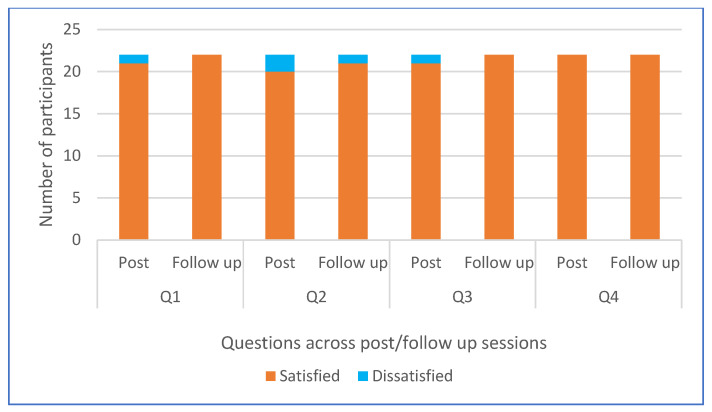
Participants’ satisfaction with four aspects (Q1–4) of the intervention at post- and follow-up sessions (n = 22). Q1 = The healthy eating learning session with the African Caribbean Eat Well guide, Q2 = Group discussion with researchers, Q3 = Cooking the selected AC recipes, and Q4 = Watching videos of AC healthier recipes and recipe cards.

At post-intervention, participants’ satisfaction with specific aspects of the intervention sessions was rated high or highest by at least 90% of participants (see Figure 3), with no significant differences in proportions of those who were satisfied/dissatisfied between subgroups of educational attainment or ethnicity. Furthermore, participants’ written feedback post-intervention included positive comments and recognition of enjoyment and new learning/experiences; “*It was very good; we never had this experience of healthy eating before*”; “*I enjoyed the sessions and have learnt a lot from them. I would love to carry on more sessions because I have learned a lot about healthy food and am willing to learn more*”; “*Really enjoyed the cooking session. You managed to get me to eat veg, and that is quite an achievement*”.

### 3.3. Potential Impact of the Intervention on Participants’ Healthy Eating Awareness, Knowledge, Skills and Behaviours

The proportion of participants who reported they were familiar with the culturally adapted African Caribbean Eat Well Guide increased from 42% (n = 8) at pre-intervention to 100% (n = 22) at both post-intervention and follow-up. Furthermore, the proportion of participants who reported at pre-intervention that they were “aware” of healthy eating guidance (42%, n = 8) and the healthier recipes (42%, n = 8) which incorporated African Caribbean (AC) foods significantly increased at post-intervention (95% n = 18) and was maintained at follow-up (*p* < 0.05) (Table 2). Likewise, the proportion of participants who reported they were “aware” of “daily allowance recommendations for salt, sugar and saturated fat” also increased significantly from pre (26%, n = 5) to post- (74%, n = 14) (*p* < 0.05) and follow-up, FU (95%, n = 18) (*p* < 0.05). However, there were no significant changes in the proportions of participants who correctly answered quiz MCQ questions testing their knowledge of daily amounts recommendations at pre-/post-time points for sugar (14%/24%), salt (33%/24%), or portions of fruit and vegetables (95%/95%) (Table 2). There were no significant differences in either awareness or correct quiz answers across sub-groups of educational attainment or ethnicity.

Participants perceived their overall cooking skills and personal importance of recipes were generally rated high (i.e., median responses 4–5 out of 5) at pre-intervention and onwards (Table 2). At post-intervention and follow-up, participants reported high levels of confidence (median 4, IQR 3–5) when using the new AC recipe resources “in day-to-day cooking”, “using healthier ingredients,” and “cooking healthier recipes within my food budget” (See Table 2). There were no differences in these levels of confidence between sub-groups according to ethnicity and educational attainment. Most participants anticipated sharing the recipe resources (n = 19, 86%) and African Caribbean Eat Well Guide (n = 18, 81%) with family and friends. At follow-up, participants’ reported changes they made after the intervention, which included: “*I buy a wider range of fruit and veg*”; “*I use less salt and sugar*”; “*I have now started to cook from scratch and buy healthier meals*”; “*I am more aware of fat foods, use low-fat coconut milk.*”

#### Participants Perceived Compliance with Eat Well Guide Recommendations

**Figure 4:**
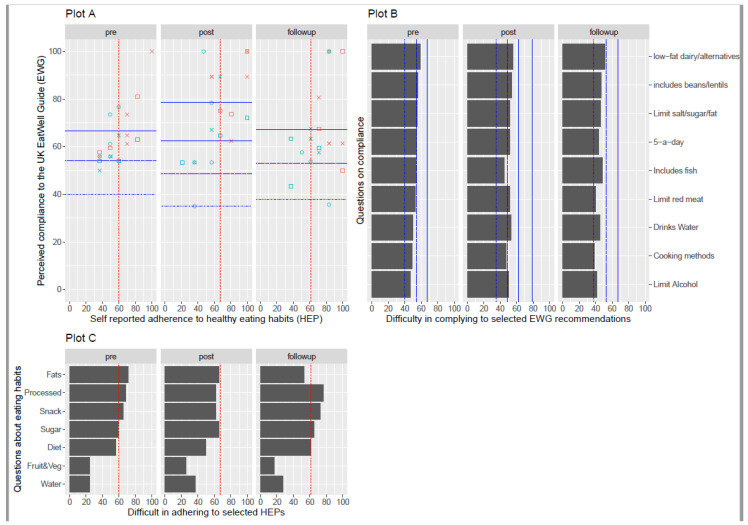
Participants number of reported healthy eating practices (HEP) and their perceived degree of compliance with the UK Eat Well guide (EWG) at pre-, post-, and follow-up. Plot **A** (Top left) represents the total scores (Rasch estimate) of HEP (low to high number of the eight aspects selected, *x*-axis) against their total scores (Rasch estimate) for compliance to EWG (from low to high, *y*-axis). Individual participant’s plots are coded red for African Caribbean and blue for other ethnicities, with educational attainment levels indicated by none (□), secondary (◦) and tertiary higher education (×). Plot **B** (top right) shows the mean estimates for items on the EWG Rasch measure (from most to least frequently chosen) indicating perceived compliance to the Eat Well Guide: Limit Alcohol—limit my consumption of alcohol; Cooking methods—use healthier cooking methods like steaming, grilling, baking, etc., instead of frying, deep frying, etc.; Drinks water—drink water over other beverages; Limit red meat—limit my consumption of red meat; Includes fish—include fish as part of my diet; five-a-day—consume five portions of fruit and vegetable every day; Limit salt/sugar/fat—limit my salt, sugar and fat consumption; Includes beans/lentils—include beans, soy, lentils, pulses and other protein sources; low-fat/alternatives—include low-fat dairy products/alternatives. Plot **C** (bottom) shows mean estimates for items on the HEP Rasch measure (from more to least frequently chosen) indicating typical eating habits: water—drinking water every day; Fruit&Veg—consuming fruit and vegetables; Diet—maintaining a balanced diet; Sugar—avoiding foods high in sugar; Snack—eating a healthy snack; Processed—avoiding processed foods and consuming whole foods; Fats—reduced fats and oils. The red dotted line indicates a significant difference (95% confidence interval) between low and high levels of adherence to HEP. Significant differences (95% confidence interval) between increasing levels of compliance to EWG are represented by different blue lines (dot, dash, two-dash and long-dash).

To evaluate perceived compliance with the Eat Well Guide recommendations via the nine-item (EWG) and seven-option multiple healthy eating practices (HEP) measures, complete data was available for 19 participants at pre-, post- and follow-up questionnaires. As determined by a Rasch rating scale model, the mean Rasch EWG estimate for all 19 participants increased from 64% (pre) to 74% (post) and 68% (follow-up) (Figure 4A). The EWG Rasch measurements suggested that participants tended to agree more with statements such as “I limit my consumption of alcohol” and “use healthier cooking methods like steaming, grilling and baking, etc.” In contrast, participants found it more difficult (tended to agree less) with statements such as “I include low-fat dairy products/alternatives” and “include beans, soy, lentils, pulses, and other protein sources” (see Figure 4B). Participants also reported an increasing number of healthy eating practices, based on the Rasch HEP measure estimate, at 57% (pre) to 64% (post) and to 73% (follow-up). Overall, fewer participants indicated their practices included “eating a healthy snack” or “avoiding processed foods and consuming whole foods” at pre-, post-, and follow-up, compared to more commonly indicated practices such as “consuming fruit and vegetables” and “drinking water every day” (Figure 4C). However, practices such as “reduced fats and oils” became increasingly likely to be reported by participants at post- and follow-up.

The mean Rasch EWG estimates for participants of African Caribbean ethnicities (n = 8) tended to be higher than the overall group means at pre-, post- and follow-up; the EWG measure was 69% (pre-), 85% (post-) and 73% (follow-up). This also contrasted with the Rasch EWG estimates for participants of other ethnicities (n = 11), which were lower than the group means at 60% (pre-), 65% (post-) and 64% (follow-up) (Figure 4A). There were no noticeable differences in Rasch EWG estimates between the participants with no secondary or tertiary higher education levels in the whole group and between ethnicities (Figure 4A).

### 3.4. Staff Views

A total of 10 staff members were recruited to take part in the in-person or remote staff intervention outline session delivered by the researchers. Most were from ethnic backgrounds other than Black Caribbean (60%, n = 6), which included White British (n = 3) and Indian/Pakistani (n = 3) ethnicities. All were female and aged 18–55 years (n = 7) or 55–65 years (n = 3). Their current roles all involved working with individuals to support mental health and long-term health conditions via assessment, signposting, group facilitation, and empowerment (i.e., role titles included Health and Wellbeing Coach, Social/Care Practitioner, Communities Officer, etc.). Populations that staff worked with include those from diverse backgrounds, underrepresented or vulnerable people, Black and Caribbean males, local communities and older people, as well as those with care plans and long-term health issues (i.e., diabetes). Half (56%) reported they possessed prior experience “working in food and nutrition,” and most were already “confident” (n = 6) or “very confident” (n = 1) about providing nutrition advice to people from different ethnic backgrounds. One staff member was already aware of the new African Caribbean Eat Well Guide, whilst others usually reported “*signpost to those with more knowledge*” when providing advice to people from different ethnic backgrounds.

**Figure 5:**
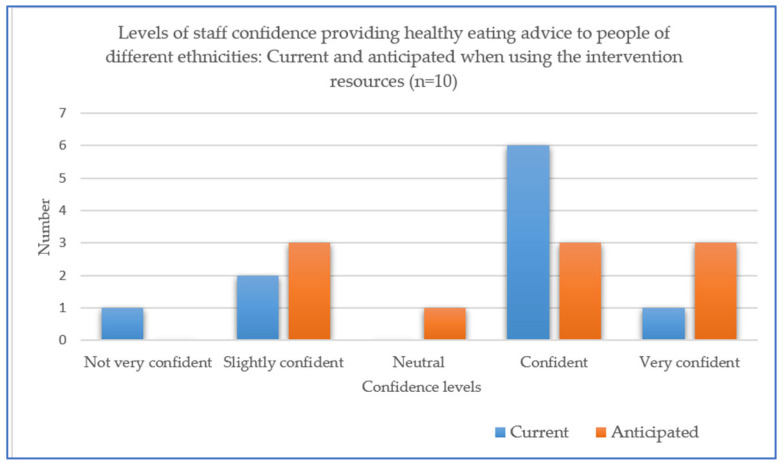
Staff (n = 10) current and anticipated levels of confidence in providing healthy eating advice to people when using the new resources.

In terms of seeking their views on how the intervention might be perceived by participants, all verbally agreed that this intervention would help participants and clients “*make healthier food choices*”, “*gain knowledge about the African Caribbean recipes*” and help people to “*try to incorporate it into their daily lives*”, based on previous interactions with people from African and Caribbean backgrounds. Two areas for possible development of the intervention were suggested by staff, including the need to reflect on vegan diets and the sensitive nature of interactions with people about diet, which might require individual (not group) approaches. Overall, slightly more staff rated their anticipated levels of confidence in providing nutrition advice to people from different ethnic backgrounds when using the new resources as “very confident” (n = 3) compared with those who selected this level when asked about their current advice provided to such people (n = 1) (Figure 5). In relation to their anticipated practical use of the new resources, staff welcomed online links and the ability to use these “via email” and anticipated the “*older community would receive it well*”. One staff member considered they would take hard copies of the resources with them as part of a household “visit pack” and another commented that “*I can now have conversations with my clients about improving diet and what to look out for*”.

## 4. Discussion

### 4.1. Key Findings

This study aimed to evidence the feasibility of a brief, new, and culturally adapted community healthy eating intervention incorporating African Caribbean traditional foods and healthier recipes from both participant and staff perspectives. Recruitment of participants, including those of African Caribbean backgrounds, was made possible via the community setting and close partnership with the community-based organisation. The majority of participants were able to attend both intervention sessions, as well as the follow-up, whilst reporting high levels of satisfaction. The collection of both verbal and questionnaire data was also evidently feasible and enabled this evaluation. In addition, the intervention was designed to feature practical food-based guidance and recipes, an approach that is widely used publicly [8], alongside cooking opportunities that have already been shown to benefit groups from vulnerable lower socioeconomic backgrounds [21]. It is therefore promising that most participants reported that following the intervention, they were highly confident using the new healthier recipes featuring African and Caribbean traditional foods at home and within budget. However, it is also possible that the intervention may have attracted participants who were already interested in cooking and recipe use, as indicated by the high levels of perceived possession of these related skills reported at pre and post-intervention time points.

In addition, also promising were our findings that participants reported pre- and post-intervention increases in levels of familiarity and awareness of the new African and Caribbean Eat Well Guide and healthier recipes resources, which persisted at follow-up. Such outcomes aligned with the objectives of the brief intervention (i.e., to raise awareness of healthy eating guidance that incorporated African Caribbean traditional and commonly consumed foods). Indeed, raising awareness and familiarity with national-level public health messaging are key first steps to health promotion campaigns, although it is possible that such campaigns may not fully support changes to relevant personal attitudes or behaviours [19]. In comparison, our findings align with those of a previous review of culturally adapted interventions [17], which suggests that culturally sensitive approaches are likely to promote the salience and acceptable uptake of intervention opportunities amongst people from diverse ethnic backgrounds.

The novel here is that this brief intervention specifically featured two new healthy eating resources, both of which were recently co-developed with people from first- or second-generation African Caribbean ethnic backgrounds [14]. As such, it is likely that the intervention is considered “culturally sensitive”, at least in terms of its superficial (“surface”) characteristics (i.e., the inclusion of specific traditional food types into healthy eating guidance) whilst incorporating cooking session leadership from staff members who were also from similar ethnic backgrounds [30]. Indeed, review evidence has highlighted that such tailoring methods and intervention design aspects may influence acceptance of diabetes prevention and management educational interventions amongst target groups [31]. Further evidence in the area of diabetes self-management education also suggests that culturally adapted interventions may better help participants reduce barriers to achieving a healthier diet and support health outcomes in this area via better recognition of needs [32]. Our findings reflect this since the objectives of this brief community intervention do appear to align with those individual-level “needs” voiced by participants at pre-intervention, including their desire to gain knowledge of healthier eating relating to specific traditional African Caribbean foods. Similarly, it is likely that the prior resource co-development work has also increased the relevance of the intervention such that most participants in the current study indicated that they intended to share the recipe resources and their learnings from the healthy eating intervention sessions with friends or family, or had done already at the follow-up (one month) time point. However, to our knowledge, there is a lack of similar studies evaluating the feasibility of culturally adapted healthy eating interventions in the community. Our findings link with those from a review of culturally appropriate health education for people in ethnic minority groups with type 2 diabetes, which found these performed better than “usual care” to support diabetes self-management outcomes [31,33].

Findings show that the participants’ objective knowledge of healthy eating was highest for daily recommended fruit and vegetable portions. Knowledge of this dietary recommendation has been found to be high amongst UK consumers previously and importantly associated with reported intakes [34]. However, participants lacked knowledge of the absolute values of specific “daily allowances” for salt and sugar, and this did not increase from pre- to post-intervention, despite considerable group discussion in the first intervention learning session on this topic and the participant’s verbally expressed surprise at learning about these recommendations. This finding suggests this brief intervention might not be effective in its current form at supporting this type of knowledge acquisition or else could be further improved by providing specific written information pertaining to these amounts. Indeed, such “daily allowances” do not currently prominently appear on either the UK or African Caribbean Eat Well Guide and may be better explained by incorporating nutrition label education [35].

It is promising that participants’ self-reported compliance with the Eat Well Guide recommendations (EWG) and practices (HEP) appeared to increase from pre- to post- (and follow-up) intervention here, inclusive of people from African Caribbean and other ethnic backgrounds, across a range of educational attainment levels. This finding is important given that achievement of a healthy balanced dietcompliant with those food-based recommendations given in the UK Eat Well Guide, remains an issue for the majority of the UK population. One study reports that just 1% of the population achieve these dietary recommendations, based on analysis of dietary intakes from a large population sample [2]. Furthermore, whilst not an objective measure of dietary intake, our use of a self-reported nine-item EWG compliance measure does provide insight into its possible future use with people from different ethnic backgrounds to relatively quickly “assess” and highlight those specific healthy eating recommendations that individuals might find more difficult (i.e., avoiding processed foods) or easier (eating fruit and vegetables) to comply with. Such an approach might be an appealing alternative to food intake records previously used to estimate compliance with the UK Eat Well Guide recommendations [36] at the population and meal levels [37,38] and a basis for future individual-level self-assessment and practical food-based improvements [39].

Furthermore, our study also sought staff views on the intervention and indicated high levels of staff confidence in using the resources in the future to support people from diverse ethnic backgrounds with healthier eating. Staff suggestions reported here can now also be used to further develop aspects of the interventions and resources (i.e., vegan-specific guidance) and indicate there is potential to integrate the intervention and resources within health professionals and community health services. Similarly, recent research, including (n = 10) the health practitioners, has also evidenced specific challenges in providing healthy eating advice in diabetes self-management education for African Caribbean communities in London, including the need for culturally adapted resources [40]. As such, the current evaluation provides insight into how to further support health professionals and community health workers, nutritionists and dietitians to enhance their cultural competence or the ability to appropriately support people from different ethnicities with healthier eating [7,11,41].

### 4.2. Strengths and Limitations

Our study sought primarily to provide people-centred opportunities to capture the voices of community staff and service users in order to evaluate the feasibility of this new intervention in an inclusive and respectful way. This was made possible and cultivated in partnership with the established Third Sector community organization, which provides services for a range of service users, including those from vulnerable and underrepresented backgrounds. Our partnership with the community organization also enabled the recruitment of people from African Caribbean ethnic backgrounds who attended the community service and lived locally. Our approach also enabled the recruitment of people with low (or no) educational attainment levels, with indications of equity (i.e., without any major differences in intervention evaluation outcomes between subgroups according to educational attainment or ethnicity). Indeed, people from such diverse backgrounds are often under-recruited in research studies conducted via other approaches (i.e., online) [14]. As such, our study now adds to the evidence on “how best to deliver” culturally-adapted diet and health educational interventions with people from diverse ethnic backgrounds, with data collection at three time points. However, the available resources and room space meant that research collaboration with only one community organization site was possible during a time frame that restricted the recruitment of more participants. Consequently, these circumstances and non-controlled study design provided a relatively small sample size, which limits the power and any conclusions around the intervention, although such circumstances are also reported in community-based evaluations of this nature [17,21,42]. Future research is required to test the actual efficacy of the intervention on outcomes associated with healthy-eating-related behaviours in a longer, larger-scale, randomized controlled study.

In addition, the intervention and cooking sessions were conducted entirely in person, and recruitment and data collection utilised existing community groups’ and data capture and was enabled by providing hard copy information and questionnaires for participant completion. Whilst inclusive and not reliant on smartphones or the internet/emails, responses provided on hard copy questionnaires were inevitably missing for some question items and therefore reduced sample size at data analysis stages. In addition, our study used limited objective measures and deliberately collected self-reported health and “body weight” assessments (i.e., would like to maintain weight, etc.) to avoid the perception of this intervention as clinical or stigmatizing [43]. Such outcomes will likely be needed in future health efficacy trials, yet our study also sought verbal data to provide a rich understanding of participant and staff perceptions of the intervention, its likely impact, and areas for improvement. Together, the questionnaire and verbal data evidenced here provide a picture of the feasibility of this culturally sensitive intervention and inform its future integration and use.

## 5. Conclusions

This study provides initial evidence to illustrate the feasibility of this new, culturally adapted healthy eating intervention that was undertaken with participants from both African Caribbean and other ethnicities in a community setting. Practical, acceptable, and relevant to staff and participants, the intervention and resources may also have potential to impact participants’ health and food and related behaviours in real life, as well as professional practice in nutrition. Future testing is warranted.

## Figures and Tables

**Table 1:** Summary of characteristics of participants who completed the pre-intervention questionnaire (n = 28) and those included in the analysis who attended both intervention sessions and completed all three questionnaires) (n = 22).

Characteristic	Participants Who Consented and Completed Pre-Intervention Questionnaires n = 28 (%)	Participants Included in Analysis Who Completed All Three Questionnaires n = 22 (%)
Gender		
Female	23 (82%)	17 (73%)
Male	4 (14%)	4 (18%)
Other	1 (4%)	1 (4%)
Age (years)		
18–55	9 (32%)	5 (23%)
55–65	10 (36%)	9 (41%)
>65	9 (32%)	8 (36%)
Ethnicity ^1^		
African Caribbean ethnicities	14 (50%)	10 (45%)
Other, including White British	14 (50%)	12 (55%)
Education ^2^		
Higher Education	10 (36%)	9 (41%)
Secondary	9 (32%)	6 (27%)
None	9 (32%)	7 (32%)
Body weight ^3^		
Happy with it	4 (14%)	3 (14%)
Lose weight	18 (65%)	15 (68%)
Gain weight	4 (14%)	3 (14%)
Prefer not to say	2 (7%)	1 (4%)
Following any dietary advice, ^4^		
Yes	5 (18%)	3 (14%)
No	23 (82%)	19 (86%)
Weekly food spend		
£10–20	3 (11%)	3 (14%)
£20–40	9 (32%)	8 (36%)
£40–60	3 (11%)	0
£60–80	4 (14%)	4 (18%)
£80–100	5 (18%)	5 (23%)
£100 and above	0	0
Prefer not to say	4 (14%)	2 (9%)

^1^ Ethnicity collapsed for summary categories were: White and Black Caribbean, White and Black African, Black African, Black Caribbean, other. ^2^ Education attainment: Higher education = University level/HNC/HND/Diploma; Secondary = NVQ, GNVQ, CSE, O-levels/GSCEs, AS/A levels, trade certificate; None. ^3^ How do you feel about your current body weight? ^4^ Have you ever received dietary advice from a health professional (i.e., are you following any dietary advice from a nurse, dietitian, doctor, or health worker, etc.)?

**Table 2:** Summary of included participants’ (n = 22) pre-, post- and follow-up responses to the questionnaire items on food-related skills and knowledge.

Measure	Questionnaire Item	Pre-	Post-	Follow-up	*p* ^$^
		Median (P25, P75) ^3^	Median (P25, P75)	Median (P25, P75)	
Recipe-related skills ^1^	Being able to cook from raw/simple ingredients	5 (4, 5)	5(4, 5)	5(4, 5)	0.862
	Following a simple recipe	5 (4, 5)	5(4, 5)	5(4, 5)	0.983
	Planning meals before shopping	4 (3, 5)	4 (4, 5)	4 (4, 5)	0.762
	Shopping for food on a budget	4 (4, 5)	4 (4, 5)	5(4, 5)	0.384
Perceived importance of ^2^	Healthy and nutritious recipe	4 (3, 5)	4 (3.25, 5)	4 (2.35, 5)	0.456
	Recipe to suit myself and whom I cook for	4 (3, 5)	4 (4, 5)	4 (4, 5)	0.875
	Recipes to suit particular dietary requirements	3 (3, 4)	4 (3, 5)	4 (3.25, 5)	0.108
	Recipe that incorporates traditional AC foods	3 (2, 4)	4 (2.25, 5)	4 (3, 4)	0.301
	A recipe recommended to me	3 (2, 4)	3 (3, 4)	3 (3, 4)	0.035
Confidence using the AC recipes ^3^	In day-to-day cooking	NT	4 (3, 5)	4 (3,4. 75)	NT
	Healthier ingredients	NT	4 (3, 5)	4 (3, 5)	NT
	Within personal budgets	NT	4 (2.25, 5)	4 (3, 5)	NT
		**% Aware**	**% Aware**	**% Aware**	
Awareness of	Healthy eating (EWG) incorporating AC foods	8 (36%)	14 (63%)	18 (81%)	0.0017
	Healthier recipes featuring AC traditional foods	8 (36%)	14 (63%)	17 (77%)	0.006
	Daily allowance of salt, sugars, and fruit and veg	5 (23%)	14 (63%)	18 (81%)	0.0003
		**% correct**	**% correct**		
Knowledge Quiz	Recommended portions of fruit and vegetables	20 (95%)	20 (95%)	NT	0.625
	Daily allowance—salt	7 (33%)	5 (24%)	NT	0.237
	Daily allowance—sugar	3 (14%)	5 (24%)	NT	0.578

^1^ Possession of recipe-related skills (1 = completely disagree, 5 = completely agree), ^2^ Importance of recipes (1 = not at all important, 5 = extremely important), ^3^ Confidence using the AC healthier recipes (1 = Not at all confident, 5 = Extremely confident). ^$^ *p* values shows results of tests of differences between pre-, post- and follow-up values using either Chi-squared (with % proportions) or Kruskal–Wallis (with medians) AC = African Caribbean, P25, P75 = 25th and 75th Percentiles. NT = Not tested.

## Data Availability

Data and materials supporting reported results can be found here: https://environment.leeds.ac.uk/dir-record/research-projects/1822/addressing-health-inequalities-in-leeds-a-pilot-healthy-eating-intervention-evaluation-with-a-third-sector-community-organisation-and-people-from-african-caribbean-communities (accessed on 1 June 2023).

## Supplementary Materials

The following supporting information can be downloaded at: https://www.mdpi.com/article/10.3390/nu15173758/s1, Participant Post session questionnaire.
