# Supplementary material for: Feasibility of a Community Healthy Eating and Cooking Intervention Featuring Traditional African Caribbean Foods from Participant and Staff Perspectives
Source: Nutrients. 2023 Aug 28;15(17):3758. doi: 10.3390/nu15173758 (PMC10490513; doi:10.3390/nu15173758)
Supplement: Supplementary file 1 [file nutrients-15-03758-s001.zip › nutrients-2532793-supplementary.pdf]

UNIVERSITY OF LEEDS

Participant Post session questionnaire

Dear participants,

Please complete a short questionnaire (10-15 minutes), which involves questions about your experience and perceptions about the sessions.

What is your name? (Used to match your responses from the pre-session questionnaires only for research purposes) *Required*

Page 2: Questions on cooking skills and preparation of food

3. After attending the sessions, to what extent do the following describe your cooking practice?

	Responses				
	Completely disagree	Disagree	Neither agree nor disagree	Agree	Completely agree
Being able to cook from raw or simple ingredients	○	○	○	○	○
Following a simple recipe	○	○	○	○	○
Planning meals before shopping	○	○	○	○	○
Shopping for food on a budget	○	○	○	○	○
	○	○	○	○	○

4. When finding recipes to cook, what is most important to you after attending the sessions?

Please select 1 answer(s) per row.

	Not at all important	Slightly important	Important	Very important	Extremely important
Finding a healthy and nutritious recipe	☐	☐	☐	☐	☐
Finding a recipe to suit myself and who I cook for	☐	☐	☐	☐	☐

Finding a recipe to suit a particular dietary requirement	☐	☐	☐	☐	☐
Finding a recipe that incorporates traditional African Caribbean foods	☐	☐	☐	☐	☐
Finding a recipe that has been recommended to me	☐	☐	☐	☐	☐

Where do you buy or obtain food from (Tick all that apply)? *Required*

- ☐ Supermarket
- ☐ Local store/shop
- ☐ Market
- ☐ Take away (on street or online)
- ☐ Food bank/pantry
- ☐ Cafe/restaurant
- ☐ Other

5.a. If you selected Other, please specify:

5.b. Has this changed since you participated in the healthy eating and cooking sessions?

Required

6. Roughly, how much money do you spend on food each week? *Required*

- £10 - £20
- £20 - £40
- £40 - £60
- £60 - £80
- £80 - £100
- £100 and above
- Prefer not to say

Page 3: Questions on the eatwell guide, dietary guidelines and awareness

7. Following the sessions, please tick the items that describe your eating habits. (Tick all that apply)

Required

- [ ] Consuming fruits and vegetables
- [ ] Drinking water every day
- [ ] Maintaining a balanced diet
- [ ] Food portion control
- [ ] Avoiding foods rich in sugar
- [ ] Reduced fats and oils
- [ ] Avoiding processed foods and consuming whole foods
- [ ] Eating a healthy snack
- [ ] Other

7.a. If you selected Other, please specify:

8. What is the recommended amount of portions of fruit and vegetable that should be consumed every day? (Tick one option) *Required*

- ○ 2 portions
- ○ 5 portions
- ○ 7 portions
- ○ Not sure

9. What is the daily recommended allowance for salt (in grams) for adults? (Tick one option) *Required*

- ○ Less than 2g
- ○ 2-4g
- ○ 4-6g
- ○ Not sure

10. What is the daily recommended allowance for free sugars (in grams) for adults? (Tick one option)

Required

- Under 10g
- 10-20g
- 20-30g
- Not sure

11. Are you aware of (State yes/ no as appropriate) the following?:

- Healthy eating guidelines which include to African Caribbean foods (Eatwell guide)
- Healthier recipes using African Caribbean traditional foods and ingredients.
- The daily allowance recommendations for sugars, salt, saturated fats and fruits and vegetables etc. with reference to African Caribbean foods.

12. Are you familiar with the African-Caribbean Eatwell guide as shown in the picture above? *Required*

- Yes
- No
- Not Sure

13. Following the session, please answer the next set of questions based on what you feel is true for you.

	Response				
	Strongly disagree	Disagree	Neither agree nor disagree	Agree	Strongly agree
In a typical week, I intend to consume 5 portions of fruit and vegetable every day.	◌	◌	◌	◌	◌
In a typical week, I intend to limit my consumption of red meat.	◌	◌	◌	◌	◌
In a typical week, I intend to include low-fat dairy products/alternatives.	◌	◌	◌	◌	◌
In a typical week, I intend to limit my salt, sugar, and fat consumption.	◌	◌	◌	◌	◌
In a typical week, I intend to use healthier cooking methods like steaming, grilling, baking, etc instead of frying, deep frying, etc.	◌	◌	◌	◌	◌
In a typical week, I intend to include beans, soy, lentils, pulses, and other protein sources.	◌	◌	◌	◌	◌
In a typical week, I intend to limit my consumption of alcohol.	◌	◌	◌	◌	◌
In a typical week, I intend to drink water over any other beverage.	◌	◌	◌	◌	◌

In a typical week, I intend to include fish as a part of my diet.	○	○	○	○	○

Page 4: Questions on your experience/perception of the sessions

14. After taking part in the activities, how confident are you of the below-listed statements?

	Responses				
	Not confident at all	Slightly confident	Confident	Very confident	Extremely confident
I am now confident about using these recipes in my day-to-day cooking.	○	○	○	○	○
I am now confident about using healthier ingredients to cook recipes.	○	○	○	○	○
I am now confident about cooking healthier recipes within my food budget.	○	○	○	○	○

15. Please rate the following based on your perceptions of the sessions: *Required*

Please don't select more than 1 answer(s) per row.

Please select at least 3 answer(s).

	Dissatisfied	Slightly satisfied	Satisfied	Very satisfied	Extremely satisfied
The healthy eating learning session with the African Caribbean Eatwell guide (Session 1)					

Group discussion session with researchers	☐	☐	☐	☐	☐
Cooking the selected healthier African-Caribbean recipes (Session 2)	☐	☐	☐	☐	☐
Watching videos of the African-Caribbean healthier recipes and recipe cards	☐	☐	☐	☐	☐

16. After attending both activity sessions, is there something that you have changed or intend to change for yourself or in your household? Please explain below. (ie what food you buy, how you cook your food etc) *Required*

17. How do you think you will use and apply the information gained from the sessions? (Tick all that apply)

- ☐ Share the new recipes with friends and family
- ☐ Share knowledge around healthy eating guidelines with friends and family
- ☐ Share knowledge on African Caribbean Eatwell guide and recommendations with friends and family
- ☐ Other

Required

17.a. If you selected Other, please specify:

18. Do you have any other feedback or comments about the sessions?

Page 5: Final page

Thank you for taking the time to take part in the post-session questionnaire! Your time is greatly appreciated.
